# Polarization Converter with Controllable Birefringence Based on Hybrid All-Dielectric-Graphene Metasurface

**DOI:** 10.1186/s11671-017-2413-1

**Published:** 2018-02-03

**Authors:** Edgar O. Owiti, Hanning Yang, Peng Liu, Calvine F. Ominde, Xiudong Sun

**Affiliations:** 1 Institute of Modern Optics, Department of Physics, Harbin Institute of Technology, Xi da zhi Road, Harbin, 150001 China; 2 Key Laboratory of Micro-Nano Optoelectronic Information System of Ministry of Industry and Information Technology, Xi da zhi Road, Harbin, 150001 Germany; 3 Key Laboratory of Micro-Optics and Photonics Technology of Heilongjiang Province, Harbin, 150001 China; 4 Collaborative Innovation Center of Extreme Optics, Shanxi University, Taiyuan, Shanxi, 030006 China; 5 Department of Physics, Jomo Kenyatta University of Agriculture and Technology, Thika Road, Nairobi, P.O.Box 62000-00200 Kenya

**Keywords:** Metasurfaces, Polarization converter, All-dielectric/graphene, Birefringence

## Abstract

Previous studies on hybrid dielectric-graphene metasurfaces have been used to implement induced transparency devices, while exhibiting high Q-factors based on trapped magnetic resonances. Typically, the transparency windows are single wavelength and less appropriate for polarization conversion structures. In this work, a quarter-wave plate based on a hybrid silicon-graphene metasurface with controllable birefringence is numerically designed. The phenomena of trapped magnetic mode resonance and high Q-factors are modulated by inserting graphene between silicon and silica. This results in a broader transmission wavelength in comparison to the all-dielectric structure without graphene. The birefringence tunability is based on the dimensions of silicon and the Fermi energy of graphene. Consequently, a linear-to-circular polarization conversion is achieved at a high degree of 96%, in the near-infrared. Moreover, the polarization state of the scattered light is switchable between right and left hand circular polarizations, based on an external gate biasing voltage. Unlike in plasmonic metasurfaces, these achievements demonstrate an efficient structure that is free from radiative and ohmic losses. Furthermore, the ultrathin thickness and the compactness of the structure are demonstrated as key components in realizing integrable and CMOS compatible photonic sensors.

## Background

Research in nanophotonics is shifting towards all-dielectric elements, particularly in designing tunable and experimentally feasible light manipulating metasurfaces [1, 2]. The primary goal is to integrate such metasurfaces into nanophotonic sensing devices. The shift of focus towards the dielectric metasurfaces is due to low radiative and ohmic losses exhibited in silicon and other dielectric materials as compared with the plasmonic metasurfaces. Consequently, special plasmonic structures using high-Q trapped mode resonances have previously been proposed to enhance transmission efficiency [2–5]. The loss reduction is achieved either through the interference between discrete electric and magnetic modes or through the symmetry breaking in the metallic elements. A weak coupling in free space is developed which enhances the loss reduction [1, 6]. Materials that show magnetic resonance such as titania (TiO_2_), silicon nitride, and germanium show good optical properties in various regions of the electromagnetic spectrum due to low losses [7–9]. In particular, they have low visible dispersion and strong electro-optic properties that enables them to be used in design of low-contrast metasurface optical elements.

Recently, graphene-based Fano resonance metasurfaces have been successfully proposed for light manipulating devices such as modulators [10–13], absorbers [14, 15], slow-light devices [16, 17], and cloaks [16, 18], as well as others. In these devices, radiative losses were mitigated as a result of strong interaction between the monolayer graphene and the confined electric field in resonant gaps. Graphene offers remarkable properties including tunable optical conductivity and high carrier mobility. This enables it to support high-Q resonant structures with suppressed radiative losses [19, 20]. On the other hand, metal metasurfaces utilize subwavelength elements to enhance electric field confinement and create abrupt changes in phase, amplitude, and polarization of the impinging light.

Split ring resonator (SRR) is a common plasmonic metasurface element because of its inductance-capacitance resonance nature that allows its flexibility in tuning optical properties. Similarly, other dielectric metasurfaces also employ the SRR as the basic metasurface unit due to its capacity for tunability and fabrication [21, 22]. Other element shapes such as “Z-slots” on silicon films have also been designed as polarization splitters [23]. However, the metal metasurfaces have high ohmic losses and low transmittance that lower their efficiency of light manipulation [24, 25].

All-dielectric meta-devices and gradient grating polarization converters, proposed by Chen et al. and Kruk et al., have shown remarkable efficiencies ∼ 99*%* [26, 27]. The structures exhibit high birefringence ratios, 0.35 and 0.9, in the terahertz and near-infrared regions, respectively. However, birefringence tunability mechanisms were not proposed. In this work, birefringence tunability and switching are demonstrated through gate voltage biasing, while structure flexibility is shown through dimension variation. Typically, metasurfaces constructed from high refractive index antennae are limited by the presence of partial back reflections due to impedance mismatch. A method to overcome this challenge is to design silicon metasurfaces with strong localized electric and magnetic Mie-type resonances so that near unity transmissions can be realized [28–30]. High contrast metasurfaces, on the other hand, have higher efficiencies but lower spatial resolution for realizing precise phase or polarization profiles along the grating lines [31, 32].

In this work, an all-dielectric metasurface with a high Q-factor based on trapped magnetic mode is shown. The proposed unit cell is composed of cross-shaped, asymmetric, rectangular dipoles made of silicon, graphene, and silica substrate. The graphene layer is sandwiched between the silicon and silica. Control of light polarization is achieved through the intrinsic properties of graphene and the dimensions of silicon, while exhibiting quarter-wave plate characteristics. Therefore, an incident linearly polarized light is converted into a circularly polarized light at a high polarization conversion ratio (PCR) in the near-infrared (> 95*%*). Moreover, the circular polarization state of the scattered light is switchable between a right-handed circular polarization (RCP), and a left-handed circular polarization (LCP) states, through an external gate voltage biasing. This dynamic control of polarization increases the degrees of freedom of the structure and can greatly impact in the CMOS photonic devices. Finite element method, using COMSOL Multiphysics, has been used to model the unit cell and analyze the performance of the metasurface.

## Methods

**Fig. 1:**
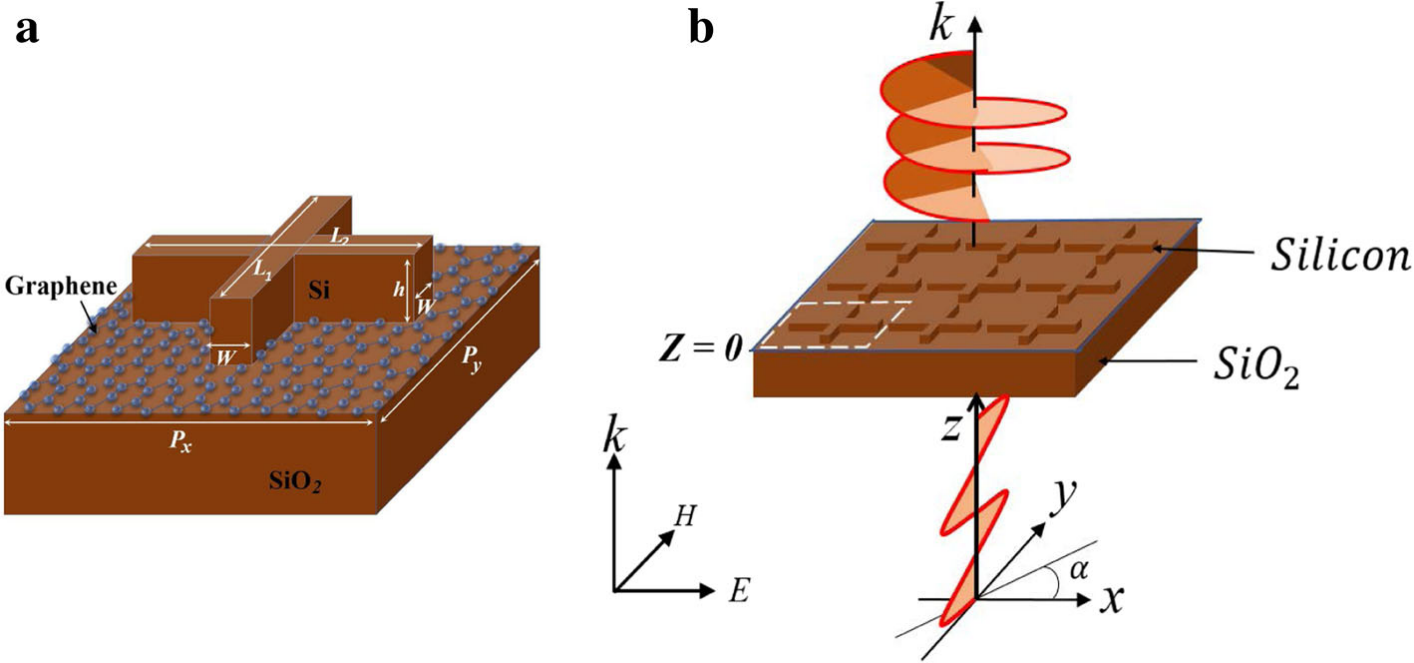
Schematic illustrations. **a.** Unit cell dimensions: *L*_1_ = 450 nm, *L*_2_ = 370 nm, *h* = 110 nm, *W* = 60 nm, *P*_*x*_ = 600 nm, and *P*_*y*_ = 560 nm. **b.** Incident linearly polarized light at an angle of polarization, *α*, converted to circularly polarized light through the structure

The schematic presentation of the structure’s unit cell is shown in Fig. 1a. It consists of a silicon cross-shaped antenna on top of a graphene layer and a silica substrate. The relative permittivity of the silicon and the silica are 12.25 and 2.25, respectively [33]. All dimensions are shown in the caption of Fig. 1a. First, to obtain an acceptable resonance, the periodicity *P*_*x*_=600 nm was fixed and *P*_*y*_ swept across several values. The internal dimensions *L*_1_=440 nm and *L*_2_=370 nm were also kept fixed but later optimized for phase tuning. The height *h*=110 nm and width *W*=60 nm were kept fixed throughout the simulations. A normally incident light from port sources, periodic boundaries, and a perfectly matched layer on the exit end were used.

The transmission properties of light were defined based on the scattered electric fields *E*_*i*_(*i*=*x*,*y*), i.e., $T_{xx} = \left |\frac {E_{x}}{E_{0}}\right |$, $T_{yy} = \left |\frac {E_{y}}{E_{0}}\right |$, *Φ*_*xx*_= arg(*E*_*x*_), and *Φ*_*yy*_= arg(*E*_*y*_), where *T*_*ii*_(*i*=*x*,*y*) are transmission coefficients and *Φ*_*ii*_(*i*=*x*,*y*) are phase components. We then defined the phase delay as $\Delta \Phi = \text {arg}\left (\frac {E_{x}}{E_{y}}\right) = \Phi _{xx}-\Phi _{yy}$ and calculated it at a distance *z*=1.2 *μ*m from the surface. A birefringent metasurface manipulates the state of polarization of the incident light by introducing a phase delay on one of the components in the transmission field. By Huygens principle, the structure creates a phase discontinuity and a phase delay between *Φ*_*xx*_ and *Φ*_*yy*_ of the transmitted light $E = E_{x}e^{i\Phi _{xx}}\hat {x}+E_{y}e^{i\Phi _{yy}}\hat {y}$. If the introduced phase delay is 90° or − 90°, an LCP or an RCP lights are produced, respectively, confirming QWP operation as illustrated in Fig. 1b. In general, the transmitted wave through the metasurface is elliptically polarized:

1 $$ \frac{x^{2}}{E_{x}^{2}}+\frac{y^{2}}{E_{y}^{2}}-2\frac{x y}{E_{x} E_{y}}\cos\Delta\Phi = \sin^{2}\Delta\Phi.  $$

Typically, the optical properties of graphene are presented through its conductivity, *σ*, characterized by both the interband and intraband transitions: *σ*=*σ*_*I*_+*σ*_*D*_, where *σ*_*I*_ and *σ*_*D*_ are the interband and intraband conductivities, respectively. A change of surface charge density, *n*_*s*_, in graphene varies the electron population in graphene and the Fermi energy, i.e., $E_{F} = \hbar \nu _{F}(\pi n_{s})^{1/2}$, where *ν*_*F*_=10^6^m/s is the Fermi velocity of electrons. We modeled graphene as a bulk monolayer of mesh cells of thickness, *δ*=1nm, and in-plane dimensions, 1 nm×1nm. The in-plane permittivity was calculated within the random phase approximations at room temperature: $\epsilon _{g}(\omega) = 1+\frac {i\sigma }{\omega \epsilon _{0} \delta } = \epsilon '+i\epsilon ''$, where *ε*^′^ and *ε*^″^ are the real and imaginary parts of the permittivity, respectively, defined as functions of the incident photon energy $E = \hbar \omega $ and *E*_*F*_:

2 $$ {}\begin{aligned} {\epsilon}^{\prime}_{\mathrm{g}} &= 1+\frac{e^{2}}{8\pi E {\epsilon}_{0} \delta}\ln\frac{(E+2|{E}_{F}|)^{2}+{\Gamma}^{2}}{(E-2|{E}_{F}|)^{2}+{\Gamma}^{2}}-\frac{e^{2}}{\pi {\epsilon}_{0}\delta}\frac{|{E}_{F}|}{{E}^{2}+\left(\frac{1}{\tau}\right){~}^{2}},\ \ \text{and} \end{aligned}  $$

3 $$ {}\begin{aligned} {\epsilon}^{\prime\prime}_{\mathrm{g}}~=&~\frac{{e}^{2}}{4 E {\epsilon}_{0} \delta}\left[1+\frac{1}{\pi}\left\{{\tan}^{-1} \frac{E-2|{E}_{F}|}{\Gamma} -{\tan}^{-1}\frac{E+2|{E}_{F}|}{\Gamma}\right\} \right]\\ &+\frac{{e}^{2}}{\pi E{\epsilon}_{0}\delta\tau}\frac{|{E}_{F}|}{{E}^{2}+\left(\frac{1}{\tau}\right){~}^{2}}, \end{aligned}  $$

where *Γ*=110 meV is energy leading to the interband transition broadening at near-infrared and *τ* is the free-carrier scattering rate. Parameter $\frac {1}{\tau }$ is assumed to be zero because of the dominance of interband transitions over the intraband transitions at near infrared [1].

## Results and Discussion

### Birefringence Control Through Fermi Energy and Structure Dimensions

**Fig. 2:**
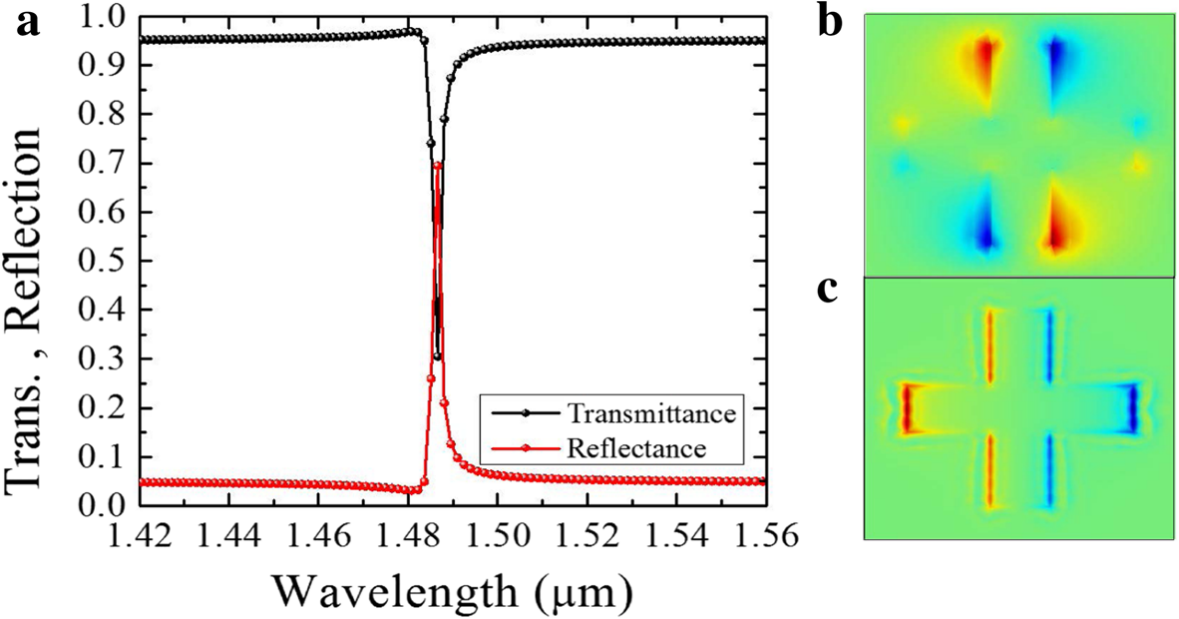
**a** Transmission and reflection for the dielectric structure without graphene. **b**, **c**. In-plane electric fields *E*_*x*_ (b) and *E*_*v*_ (c), calculated at the resonance wavelength *λ* = 1.49 *μ*m

**Fig. 3:**
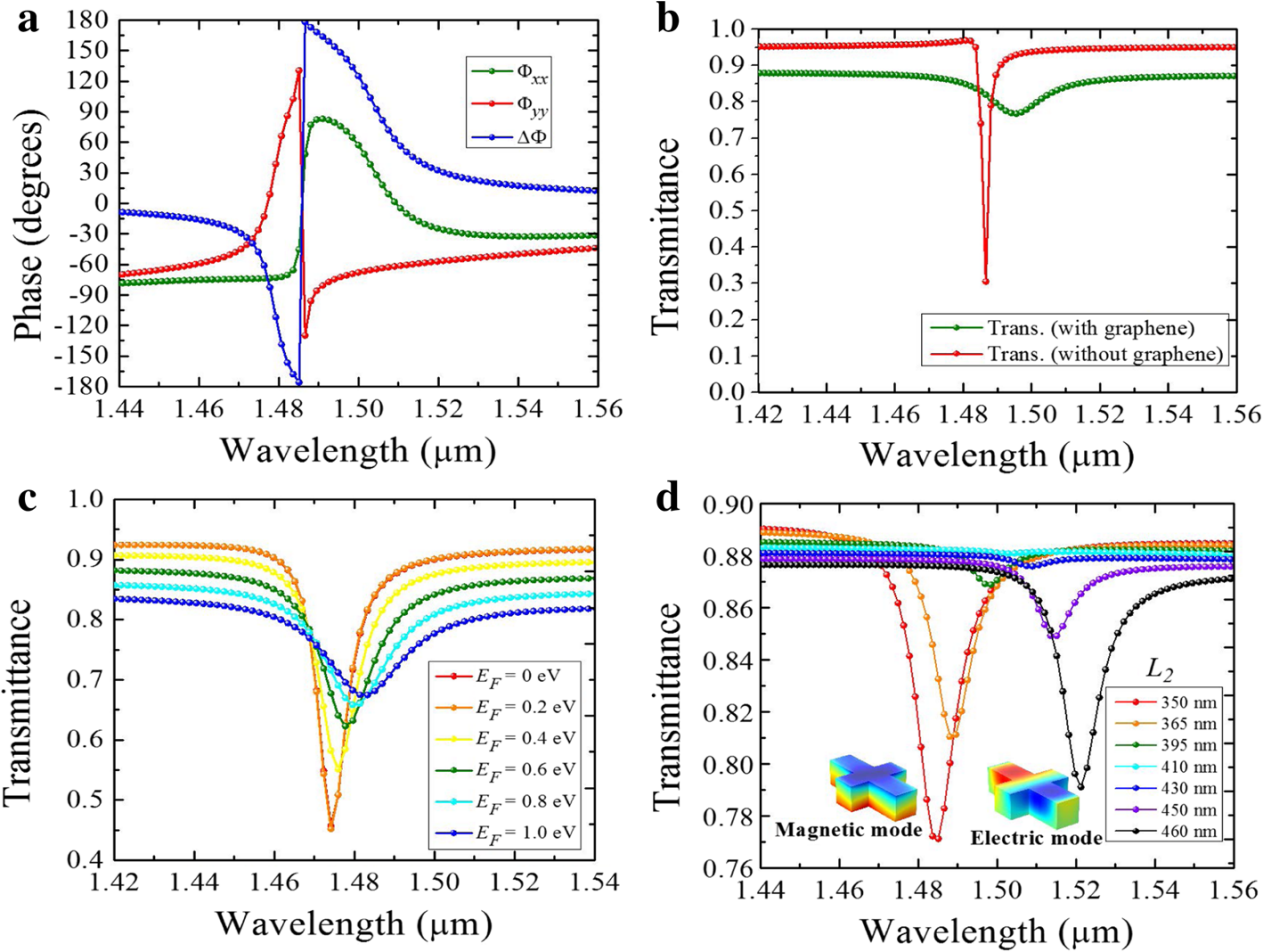
**a** Phase components and retardation of an all-dielectric metasurface without graphene. Transmittance plotted as a function of wavelength for *L*_1_ = 440 nm, *L*_2_ = 370 nm, and *W* = 60 nm, for **b** structure without graphene and with graphene (*E*_*F*_ = 0.8 eV), **c** varying Fermi energy, and **d** varying *L*_2_ from 350 to 450 nm. Symmetry breaking at *L*_2_ = 410 nm splits two dominant modes: magnetic and electric

First, the all-dielectric metasurface without graphene layer was simulated and obtained the transmission spectra shown in Fig. 2a. The structure was illuminated by an incident linearly polarized light (LP), at an angle of polarization, *α*, as illustrated in Fig. 1b. The transmittance results in Fig. 2a show a narrow resonance with high Q-factor. This is attributed to the excitation of trapped magnetic modes. There is strong in-plane electric field at the resonance wavelength *λ* = 1.49 *μ*m along the edges of the antenna (Fig. 2b). The in-plane electric fields are anti-parallel and cause a destructive interference effect between the electric and magnetic dipole responses. The components of the incident LP light at an angle of polarization, *α* = 48°, cause a weak coupling between the trapped electromagnetic modes and the free-space light. Additionally, strong field penetration into the silicon dipole results in a sharp phase shift and enhanced coupling between the incident plane wave and the circulating displacement current. A strong magnetic resonance and an abrupt phase change occurs at the resonance wavelength as shown in Fig. 3a, b. The magnetic dipole mode is influenced by the circular displacement current more than the electric mode, which is mainly due to coupling between the neighboring antenna dipoles. In adddition, Kirshav et al. demonstrated that the magnetic resonance is influenced by the dimension and shape of the structure [34]. For example, in our structure, the lateral dimensions and the wavelength of the incident light can be related through $L_{i}(i~=~1,2)\approx \frac {\lambda }{n_{\text {si}}}$, where *L*_*i*_≈440 nm and *n*_si_ = 3.5.

When the graphene layer is inserted between the substrate and the nanoantenna, the circulating displacement current inside the silicon antenna is reduced and the surface electric field is enhanced. This corresponds to the condition where polarization of the incident electric field is anti-parallel at the opposite boundaries of the nanoantenna that gives rise to a weak coupling with the circulating displacement currents within the element. Graphene introduces an enhanced conduction in the surface between the silicon and silica substrate. A stronger coupling with the in-plane electric field occurs in comparison to the coupling with displacement current within the element. Because of this effect, the anti-parallel electric fields, which would otherwise cause destructive interference on the surface, are reduced, and the Q-factor significantly drops, as shown in Fig. 3b. The resonance wavelength also shifts slightly from *λ* = 1.49 *μ*m to *λ* = 1.5 *μ*m due to the reduced penetration into the silicon. In Fig. 3c, the effect of varying the Fermi energy of graphene is shown. For an undoped graphene (*E*_*F*_ = 0 eV), there is a strong resonance at *λ* = 1.5 *μ*m which diminishes as the doping level is increased. The interband transition dominates when the Fermi level is low and graphene exhibits dielectric characteristics with a larger *ε*^′^. However, when *E*_*F*_ is increased, several interband transition channels are blocked; the intraband transitions then cause graphene’s inductive response and decreases its absorption [1, 20]. It is worth noting that with graphene under-layer and proper dimensions of the silicon structure, the magnetic and electric dipole modes can be enhanced in strength, leading to a high scattering efficiency [34]. The silicon antennae exhibit coupled resonances from two close wavelengths around resonance as shown in Fig. 3d. At *λ* = 1.48 *μ*m, the antenna shows coupling of induced magnetic dipoles, while at *λ* = 1.52 *μ*m, the coupling is between the electric modes. The two modes occur when the symmetry of the antenna changes from *x* to *y* orientations at *L*_2_≈410 nm. The dimension *L*_2_ was swept through a range of values between 350 and 480 nm while keeping *L*_1_ fixed at 440 nm.

**Fig. 4:**
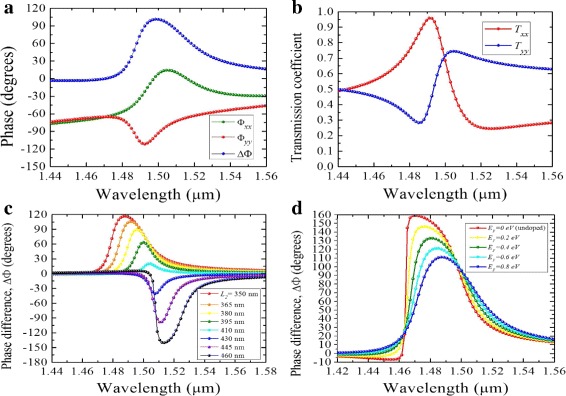
**a**Phase components and retardation of an all-dielectric/graphene metasurface and **b** the corresponding transmission coefficients *T*_*xx*_ and *T*_*yy*_. Phase retardation plotted as a function of wavelength for *L*_1_ = 440 nm, *L*_2_ = 370 nm, and *W* = 60 nm, for **c** varying Fermi energy and **d** varying *L*_2_ from 350 to 450 nm

The graphene effect is beneficial for tuning the phase components and the phase retardation of the transmitted electric fields. Firstly, the components of the incident LP light are decomposed into the orthogonal arms of the silicon antenna. Each dipole resonance imprints a different phase pattern on the scattered light. Specifically, near the resonance, each dipole resonance shifts the phase of the incident electric field in the range [− *π*,*π*]. With proper dimensions of the antenna, a 90° phase difference is obtained as shown in Fig. 4a. The corresponding transmission coefficient is shown in Fig. 4b. It is noticeable that the intersection point *T*_*xx*_ = *T*_*yy*_ occurs near the resonance, defining an ideal QWP condition. Additionally, by sweeping through different values of the length *L*_2_ while keeping *L*_1_ fixed (*L*_1_ = 440 nm), the resonance amplitudes associated with different electric and magnetic modes can be varied. An acceptable phase bandwidth range within ± 10° was obtained when *L*_2_ = 365 nm for RCP, and *L*_2_ = 450 nm for LCP, as shown in Fig. 4c. Secondly, in Fig. 4d, by varying the Fermi energy of graphene, the phase bandwidth changes accordingly. At *λ* = 1.48 *μ*m, the undoped graphene (*E*_*F*_ = 0 *eV*) causes high penetration of electric fields into the silicon dipoles and a large phase difference between the *x* and *y* components of scattered light (≈150°) occurs. However, as *E*_*F*_ approaches 0.8 *eV*, the in-plane properties (*ε*_*x*_ = *ε*_*y*_) increase the surface conductivity of graphene, resulting in a reduced penetration into the silicon and a *ΔΦ*≈90° at *λ* = 1.49 *μ*m.

**Fig. 5:**
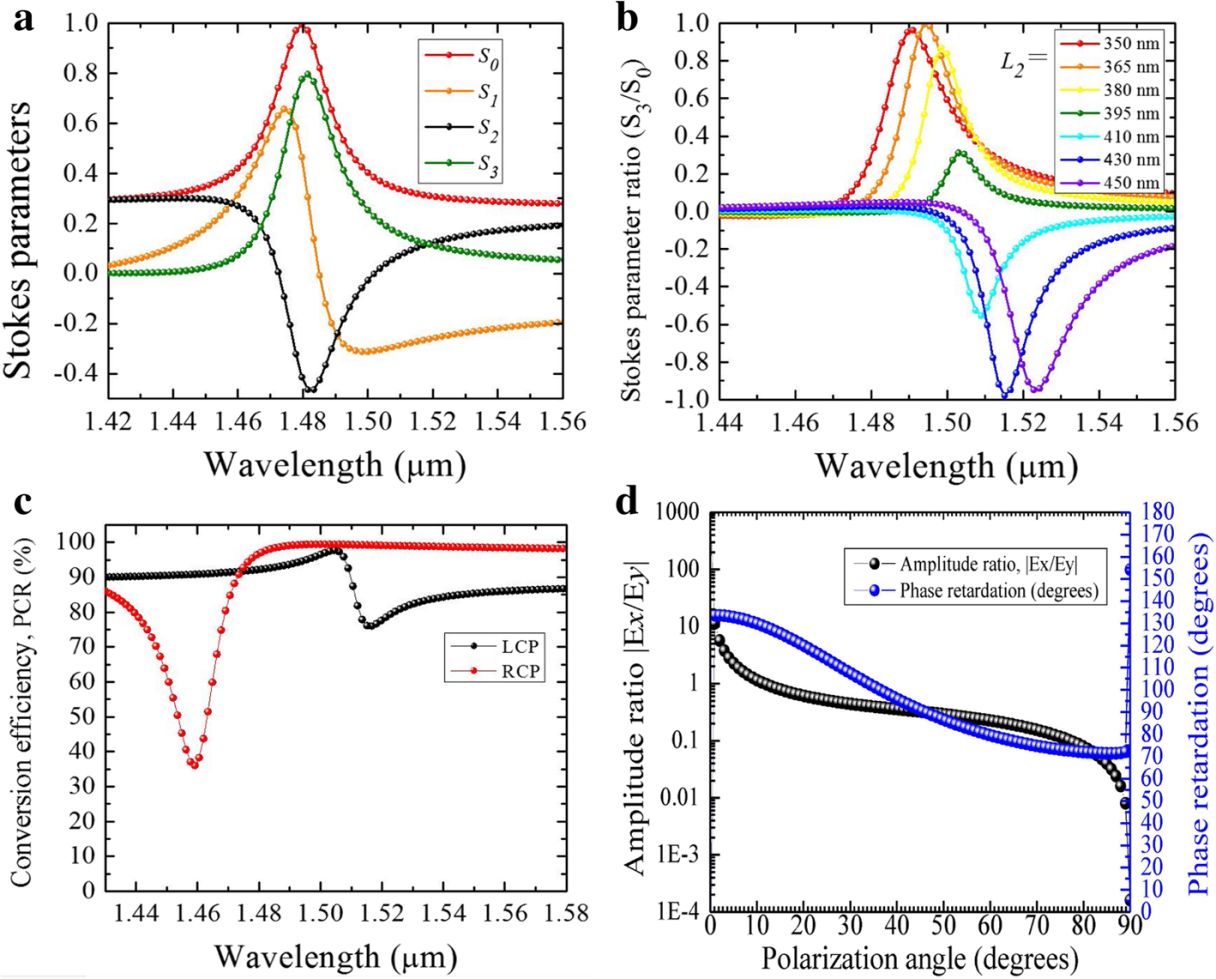
**a** Stokes parameters variation against the wavelength for an incident angle of polarization *α* = 48°. **b**. Stokes parameter ratio (*S*_3_/*S*_0_) variation as a function of *L*_2_ at *α* stated in **a**, **c** polarization conversion ratio calculated for an incident linearly polarized light. **d**. Ratio of amplitudes and phase difference at the wavelength *λ* = 1.5 *μ*m as a function of polarization angle

The calculated Stokes parameters and polarization ellipse dimensions for the hybrid structure with *L*_1_ = 450 nm, *L*_2_ = 370 nm, and *W* = 60 nm are shown in Fig. 5a, b. It is noted that away from the resonance wavelength, the polarization of transmitted light remains unchanged from that of the incident light. However, near the resonance, the polarization state changes to circular for an incident LP light. At *λ* = 1.5 *μ*m, the Stokes parameter ratio |*S*_3_/*S*_0_|≈ ± 1, where a + 1 value indicates a perfect RCP and a − 1 indicates a perfect LCP output. Here, *S*_0_ = |*E*_*x*_|^2^+|*E*_*y*_|^2^ and *S*_3_ = 2*E*_*x*_*E*_*y*_ sin*ΔΦ* are the Stokes parameters. The degree of transmission intensity is determined by *S*_0_, i.e., a value > 50*%* is acceptable. Figure 5c shows PCR efficiency calculated from the transmission coefficients:

4 $$ \text{PCR}~=~\frac{T_{yx}^{2}}{T_{yx}^{2}+T_{xx}^{2}},  $$

where *T*_*yx*_ and *T*_*xx*_ are cross and co-polarization terms, respectively. Within the wavelength range *λ* = 1.48 *μ*m and *λ* = 1.51 *μ*m, the efficiency is ≈96*%* for RCP and ≈90*%* for LCP outputs. However, at *λ* = 1.52 *μ*m, the efficiency slightly drops to ≈80*%* for LCP. As shown in Fig. 5d, the structure is insensitive to the angle of polarization of the incident LP light. Acceptable amplitude ratio *E*_*x*_/*E*_*y*_≈1 and phase shift *ΔΦ*≈90° are obtained in a wide range. When *α* = 48°, an accurate QWP condition is obtained

**Fig. 6:**
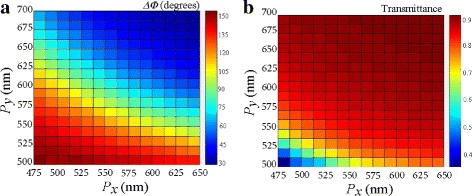
**a**–**b** Variation of periodicities *P*_*x*_ and *P*_*y*_ at *λ* = 1.5 *μ*m. **a** Transmission phase and **b** transmittance

Additionally, the transmission phase profile defining the form birefringence was calculated as a function of the periodicities *P*_*i*_(*i* = *x*,*y*) at the wavelength *λ* = 1.49 *μ*m. In Fig. 6a, tunable phase retardation of the structure is obtainable along the diagonal where the two periodicities show an inverse relation. It is also worth noting that the phase retardation (*ΔΦ*≈90°) occurs in the region where the transmittance is above 80%, as shown Fig. 6b. Silicon and silica have low dispersion and relatively high refractive indices, hence supporting low absorption in the shorter wavelengths [8]. Similarly, the phase output can be controlled through an external gate voltage.

### Birefringence Switching Through Gate Voltage Biasing

**Fig. 7:**
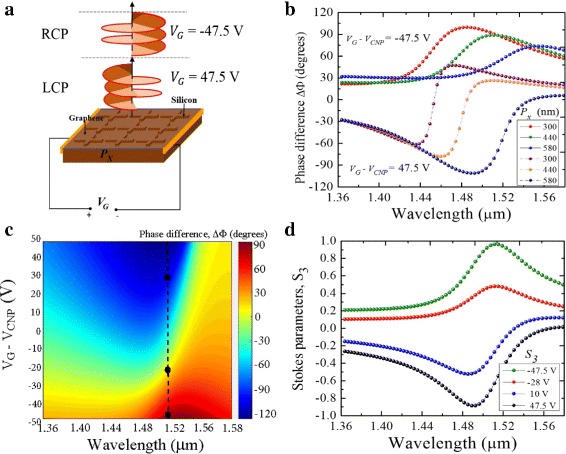
**a** Schematic illustration of the silicon/graphene switching of the polarization state through gate voltage biasing. **b.** Simulated phase difference as a function of gate voltage biasing. **c.** Phase difference shown as function of periodicity *P*_*x*_ and gate voltage. **d.** Stokes parameter *S*_3_ spectra showing the two states of circular polarization defined by the different gate voltages

Application of gate voltage bias across the *y*-planes of the silicon/graphene structure was designed as shown in Fig. 7a. By switching the gate voltage between a forward biasing value and a reverse biasing value, the incident LP light is dynamically converted into RCP and LCP states of the scattered lights, respectively. The bias voltage controls the Fermi velocity of electrons, *ν*_*F*_, and switches the direction of flow of electrons. Additionally, the bias voltage changes the carrier density of graphene which in turn leads to a change in its electrical conductivity and permittivity. In this configuration, the structure forms a quasi-parallel plate capacitor model with an electrostatic capacitance per unit area, *C*, defined as *C* = *ε*_*si*_*ε*_0_/*P*_*x*_, where *ε*_*si*_ is the dielectric permittivity of silicon. The Fermi energy, $E_{F}~=~\hbar \nu _{F}\sqrt {\pi n_{s}}$, is also modulated. The charge density (*n*_*s*_) and the electrostatic capacitance per unit area (*C*) scale the Fermi energy through the gate voltage, that is, *n*_*s*_ = *CV*_*G*_/*e*. Consequently, an increment in *P*_*x*_ decreases both the carrier concentration in graphene and the capacitance per unit area. As a result, as shown in Fig. 7b, the position of the phase retardation is red-shifted, consistent with perturbation theory in the mid-infrared [35].

At *λ* = 1.5 *μ*m, the two states of circular polarization can be encoded as two binary states, 0 and 1. The logic state 0 corresponds to the reverse voltage − 47.5*V* while the logic state 1 corresponds to the forward voltage 47.5*V*, as shown in Fig. 7c. A very little change in the phase retardation, *ΔΦ*≈0°, can be observed when the gate voltage is at − 25 *V* (along the black dotted line of the figure). This observation shows a non-linear response in the phase change at − 47.5, − 25, and 47.5 *V*, attributed to a variation in capacitive coupling as graphene becomes more conductive because of a change in the carrier density and gate voltage. In comparison to other wavelengths in the near-infrared, 1.5 *μ*m shows the optimum point for switching the circular polarization states of the scattered light.

In Fig. 7d, the stokes parameters *S*_3_ illustrates the degree of circular polarization as a result of the gate voltage biasing. The − 1 and 1 limits denote the ideal polarization conversions from a linear state to LCP and RCP states, respectively. Between the wavelengths *λ* = 1.49 *μ*m and *λ* = 1.52 *μ*m, the degree of circular polarization approaches unity (> 90*%*) for both states, confirming the most appropriate operation region of the structure as a QWP.

**Fig. 8:**
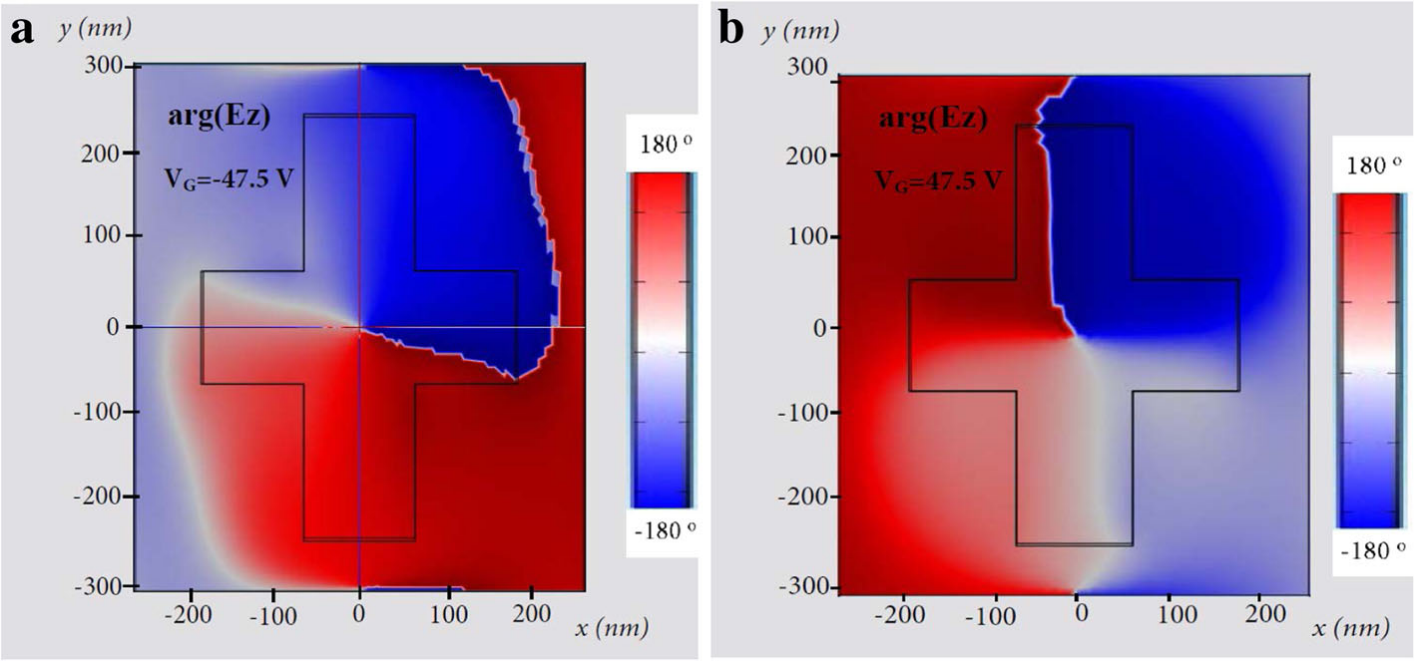
Phase map of electric field component *E*_*z*_ through the silicon/graphene cross-shaped structure at *z* = 0 calculated at the design wavelength *λ* = 1.5 *μ*m, **a** when the gate voltage is *V*_*G*_ = − 47.5 *V*, and **b** when the gate voltage is *V*_*G*_ = 47.5 *V*

Figure 8a, b shows the phase distribution of the *z* component of the electric field, calculated at the design wavelength *λ* = 1.5 *μ*m at *z* = 0. The distribution shifts as the voltage is reversed from 47.5 to − 47.5 *V*. The change in electrical conductivity and carrier density of graphene results in a rotation of the trapped magnetic mode around the silicon structure.

## Conclusions

In summary, birefringence controllability of a hybrid silicon/graphene metasurface polarization converter has been numerically designed. Trapped magnetic modes and high Q-factors are modulated by integrating graphene and silicon. Two configurations of the hybrid structure have been shown, one with a gate voltage bias and the other without. In the voltage-biased structure, birefringence performance is shown through reversal of the gate voltage. From an incident LP light, a reverse bias voltage (- 47.5 V) produces an RCP output and a forward bias voltage (47.5 V) produces an LCP output. Hence, a dynamic switching performance is achieved. For the free-space configuration, QWP performance is shown through manipulation of the dimensions of silicon and the Fermi level of graphene. In both designs, a more stable and broader bandwidth is obtained than in structures without graphene. The designs show higher degrees of polarization conversions (>96*%*) in the near-infrared (*λ* = 1.45 to 1.54 *μ*m). Unlike in plasmonic metasurfaces, these achievements demonstrate high efficiency devoid of radiative and ohmic losses. Additionally, the structures are compact and have an ultrathin thickness, appropriate for compatibility and integration with CMOS and photonic devices. Meanwhile, graphene is feasible and can be grown using chemical vapor deposition on the substrate while the silicon structure can be fabricated using standard lithographic methods.
